# Modeling elucidates how refractory period can provide profound nonlinear gain control to graded potential neurons

**DOI:** 10.14814/phy2.13306

**Published:** 2017-06-08

**Authors:** Zhuoyi Song, Yu Zhou, Mikko Juusola

**Affiliations:** ^1^ Department of Biomedical ScienceUniversity of SheffieldSheffieldUnited Kingdom; ^2^ School of EngineeringUniversity of Central LancashirePrestonUnited Kingdom; ^3^ State Key Laboratory of Cognitive Neuroscience and LearningBeijing Normal UniversityBeijingChina

**Keywords:** *Drosophila*, fly photoreceptor, large dynamic range, neural adaptation, quantal sampling, quantum bump, stochasticity, vision

## Abstract

Refractory period (RP) plays a central role in neural signaling. Because it limits an excitable membrane's recovery time from a previous excitation, it can restrict information transmission. Classically, RP means the recovery time from an action potential (spike), and its impact to encoding has been mostly studied in spiking neurons. However, many sensory neurons do not communicate with spikes but convey information by graded potential changes. In these systems, RP can arise as an intrinsic property of their quantal micro/nanodomain sampling events, as recently revealed for quantum bumps (single photon responses) in microvillar photoreceptors. Whilst RP is directly unobservable and hard to measure, masked by the graded macroscopic response that integrates numerous quantal events, modeling can uncover its role in encoding. Here, we investigate computationally how RP can affect encoding of graded neural responses. Simulations in a simple stochastic process model for a fly photoreceptor elucidate how RP can profoundly contribute to nonlinear gain control to achieve a large dynamic range.

## Introduction

Refractory period (RP) determines an excitable membrane's recovery time (Hodgkin and Huxley 1952b). During RP, the membrane patch cannot respond to external stimuli, no matter how strong these are. Thus, RP can restrict a neuron's firing patterns (Adrian & Zotterman, 1926) and information transmission capacity; or, how many different stimulus patterns it can encode as different in a unit of time (Juusola et al. 2007). But it remains less clear whether or how RP exchanges this loss in capacity to some other encoding benefits.

The neural encoding of sensory stimuli is most commonly investigated for action potential responses, using either the classic “rate code” or “time code” concepts (Gautrais and Thorpe 1998; van Rullen and Thorpe 2001; Brette 2015), each of which considers the role of RP differently. In the “rate code” (Adrian & Zotterman, 1926), RP would limit a neuron's average firing rate and, thus, its encoding performance. Conversely, in the “time code,” the precise spike timing relations convey the message, and RP would affect spike timing reliability (Berry and Meister 1998; Avissar et al. 2013).

However, many sensory neurons, such as retinal photoreceptors, crustacean stretch receptors and vertebrate hair cells (Roberts and Bush 1981), use graded potentials to encode fast and large stimulus changes. This requires powerful adaptation, which continuously adjusts their sensitivities to environmental changes. In fly photoreceptors, refractory quantal sampling of light changes facilitates encoding of salient stimuli over the full diurnal range (Song et al. 2012; Juusola et al. 2015). And in all neurons, graded signals further communicate quantal transmitter release during synaptic transmission (Juusola et al. 1996, 2007; Debanne et al. 2013). Refractoriness could thus contribute importantly to adaptive regulation of quantal events in small signaling compartments (Stevens and Wang 1995; Hardie 2012). Meaning that, instead of RP just adapting action potential firing patterns, another type of RP may have already adapted the subthreshold signals that drive them.

In this study, we use a simplified fly photoreceptor model to elucidate how RP can provide generic nonlinear gain control to graded potential neurons. A fly photoreceptor is a classic graded potential system for studying neural representation of environmental signals (Weckstrom 1989; van Hateren 1997; Juusola and Hardie 2001a,b; Song et al. 2012). It can transduce vast environmental light intensity changes (~10 log units; photons/sec per *μ*m^2^) into graded macroscopic responses within its limited (40–65 mV) output range (Juusola and Hardie 2001a,b; Juusola et al. 2016), achieving a much larger dynamic range than man‐made sensors (Song et al. 2012; Song and Juusola 2017). Owing to *Drosophila* genetics and accessibility of electrophysiological experiments (Hardie and Juusola 2015), much is known about phototransduction in microvillar photoreceptors, enabling mechanistic investigations into adaptation.

A detailed biophysical fly photoreceptor model can accurately mimic how its real counterparts encode light stimuli (Song et al. 2012; Song and Juusola 2014; Juusola et al. 2016). A fly photoreceptor's light sensor, the rhabdomere, is composed of 30,000–90,000 microvilli (photon sampling units), each of which absorbs photons stochastically. Each microvillus contains a full G‐protein‐coupled‐receptor (GPCR) signaling pathway, capable of adaptively transducing absorbed photons into quantum bumps (QBs: single photon responses). Because of local negative feedbacks inside its GPCR signaling pathway, the microvillus stays refractory (inactivated) for about 100 msec after producing a QB, during which it cannot respond to new photons (Hardie 2012). Finally, QBs from all microvilli sum up the macroscopic light‐induced‐current (LIC) response. Thus, through the “stochastic adaptive quantal information sampling” scheme, the model generates realistic neural responses to environmental light changes. Simulations have shown that RP (1) can intrinsically arise from neural sampling of quantal events, and (2) contributes to achieving a large dynamic range (Song et al. 2012; Song and Juusola 2014; Juusola et al. 2015). Here, we reduce the biophysical photoreceptor model into a generic stochastic process model with only four parameters, and derive some theoretical results from this mathematical analysis.

Despite its simplicity, the new model makes a useful tool for characterizing how RP affects neural sampling and summation of quantal events at different light (stimulus) conditions. Because photon arrival rate to an individual microvillus changes proportionally with light intensity, its average inter‐photon‐intervals can either be longer or shorter than the average RP. In dim conditions, RP cannot restrict encoding; as photons arrive sparsely, the inter‐photon‐intervals are much longer than their refractory periods and all photons are transduced to QBs (100% quantum efficiency, QE). But with brightening, more photons are lost to RPs and the QE reduces nonlinearly. Thus, by reducing sensitivity in proportion to background light intensity, RP represents a fast automatic adaptation mechanism, which enables fly photoreceptors to maintain vision over a large dynamic range (see also: Juusola et al. 2015; Song et al. 2012).

Our new results define the theoretical bounds for such adaptation, and show how it depends on the statistical RP properties. Because refractoriness is likely a ubiquitous biophysical phenomenon, affecting different stages of neural signaling, from single channel dynamics to synaptic transmission (Stevens and Wang 1995), these results also shed new light on how it may contribute to adaptation in other graded potential neurons.

## Model

**Figure 1:**
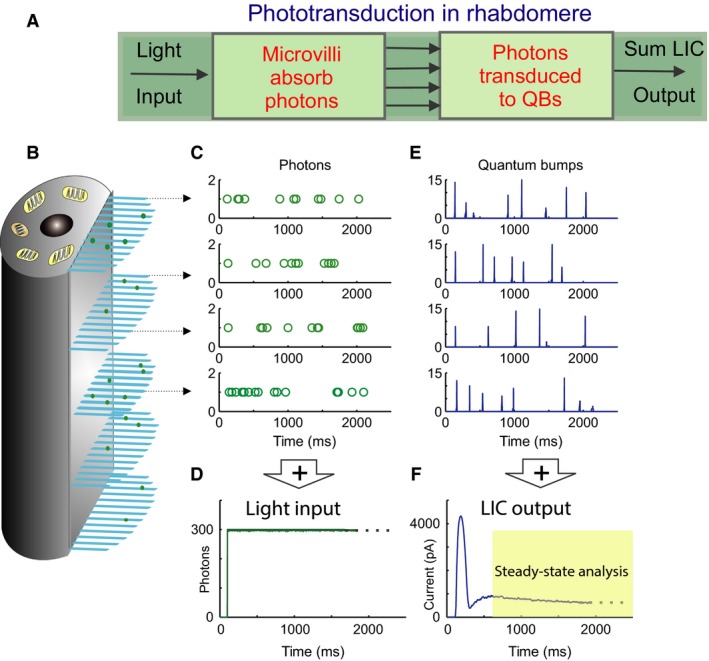
Fly phototransduction model schematic. (A) The phototransduction takes place in the rhabdomere, which transduces light input (a dynamic flux of photons) into macroscopic output, light‐induced current (LIC). (B) The rhabdomere contains 30,000 microvilli (blue bristles), acting as photon sampling units. (C) Photons are randomly distributed over the 30,000 microvilli. Because of the large microvillus population, each of them will only absorb a photon sequence, which can be approximated by a Poisson point process (each row of open circles indicate a photon sequence absorbed by a single microvillus over time). (D) The light input (green trace) can be reconstructed by adding up all the photons distributed across the 30,000 microvilli. (E) The successfully absorbed photons in each microvillus are transduced into QBs (a row of QB events). In each microvillus, the success of transducing a photon into a QB depends upon whether the microvillus is in its refractory state. The photons hitting a refractory microvillus cannot evoke QBs, but will be lost. (F) QBs from all the microvilli integrate the dynamic macroscopic LIC.

We constructed a simple stochastic process model of microvillar phototransduction, having only four biophysical parameters. The model maps the light input, *I*(*t*) (a dynamic influx of photons) into the graded macroscopic LIC, *C*(*t*) (Fig. 1A). Here, for simplicity, we focus upon steady‐state responses to light intensity steps of known absorbed photon counts (#photons/sec).

**Figure 2:**
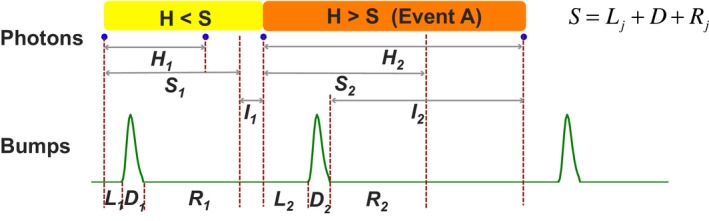
Schematic of photon sequence and QB sequence in a microvillus. *H* denotes the photon arrival intervals. *L* is the latency in converting a photon to a QB and *R* is the refractory period after a QB. *D* is the bump duration and *S* the minimum inter‐QB‐intervals, i.e. any photons that arrive during *S* will be lost. *S* can be calculated as the summation of *L*,* D* and *R*. *I* is the waiting time for the next photon arrival. Depending the relationships between *H* and *S*, inter‐QB‐intervals, *T*, can be approximated by different quantities. If *Hk*>*Sk*, then *Tk* = *Hk* or *Tk Lk* + *Dk* + *Ik*, otherwise, *Tk* = *Sk*+*Ik*.

In the model, incoming photons are stochastically distributed to 30,000–90,000 microvilli (photon sampling units; Fig. 1B). With this many sampling units, each microvillus will only absorb a photon sequence, which can be approximated by a Poisson point process (Fig. 1C). The absorbed photons in each microvillus are transduced into a sequence of QBs (Fig. 1E). The QB generation success depends upon whether the microvillus is in a refractory state, as all the photons absorbed by refractory microvilli will be lost. QBs from all the microvilli integrate the dynamic macroscopic LIC (Fig. 1F), as governed by four biophysical parameters (Juusola and Hardie 2001; Song et al. 2012): (1) the total number of microvilli; (2) the sample size (QB waveform); (3) the latency distribution (time delay from a photon absorption to a QB emergence (Fig. 2)), and (4) the refractory period distribution (microvillus recovery time after a QB). Next, we formulate these terms mathematically.

As light information is quantal, communicated by stochastic photon arrivals, the phototransduction begins with photon absorptions within the microvillus population. We assume that all incoming photons are absorbed, and all microvilli absorb their photons independently with equal probabilities. Under this assumption, photon absorptions by the nth microvillus are modeled as a Poisson point process (Eq. 1).

(1) $$I_{n}\left(t\right)=\sum_{k=1}^{\infty }\delta_{n}\left(t-t_{k}\right)$$

where *t*_k_, *K* = 1,2 … . are the successive photon arrival instants, and *δ*(*t* ‐ *t*_*k*_) is Dirac's delta function that is zero, except at time *t*_k_.The event time ensemble {*t*_k_} is a Poisson point process (Song et al. 2016).

Conversely, we assume that each microvillus transduces its absorbed photons to QBs, independent of the other microvilli. The QB sequence can be written as:

(2) $$C_{n}\left(t\right)=\sum_{j=1}^{\infty }hB_{n}\left(t-b_{j}\right)$$

where *B*(*t*) is the QB waveform, in which maximum value is normalized to one. For simplicity, we assume that every QB has a uniform waveform, with a fixed amplitude *h* and duration *D*. {*b*_j_} denotes the QB recurrence times, with this ensemble exhibiting stochastic variations.

From Eqs (1), (2), the transformation from {*t*_k_} to {*b*_j_} is a key factor that determines the relationship between *I*_*n*_(*t*) and *C*_*n*_(*t*). Two rules govern the transformation from {*t*_k_} to {*b*_j_}:

1. In a microvillus, a photon evokes a QB after a short delay, *L* (latency), which represents the molecular phototransduction cascade reaction time.
2. Following a QB, the microvillus is inactivated for a RP. During this dead‐time, *R*, another QB cannot be evoked, even though the microvillus may absorb other photons then. Hence, a photon can only trigger a QB when the microvillus is not refractory.

To reflect the stochasticities, inherent to the phototransduction cascade, both the latencies and dead‐time are independent and identically distributed (i.i.d.) random variables, defined by their own distributions. For mathematical convenience, we assume that both *L* and *R* follow gamma distributions. This is also suggested by the experimentally derived latency distributions (Juusola & Hardie, 2001a,b). In this way, {*b*_j_} is a stochastic renewal process, rectified from the Poisson point process {*t*_k_} with variables *L* and *R*. *C*_*n*_(*t*) is then a shot noise process, formed by convolving {*b*_j_} with *B*(*t*).

Lastly, summation of QBs from all the microvilli produces the macroscopic LIC. Mathematically, this summation represents a superposition from many shot noise processes *C*_*n*_ (*t*):

(3) $$C\left(t\right)=\sum_{n=1}^{N}C_{n}\left(t\right)$$

where *N* is the photoreceptor's total microvillus count (the number of sampling units).

## Results

We define a photoreceptor's input‐output gain as the ratio between the *expected C*(*t*) and *I*(*t*), denoted *E*(*c*) and *E*(*I*), respectively, and ask how this ratio changes at different light intensity levels. For analytical simplicity, we use the quantum efficiency (QE) to approximate a normalized version of this ratio at steady state. QE measures the proportion of photons that are successfully transduced to QBs (the total QB number, N_QB_, divided by the total number of absorbed photons,N_ph_, over a time period of *Tt*; Eq. 4). Because one photon can maximally evoke one QB, the QE is always ≤1 and acts as a gain factor between a photoreceptor's light input and neural output.

Assuming that all microvilli absorb and transduce photons independently with equal probabilities, both the photon arrival and QB rates will be the same for every microvillus. So we can approximate a photoreceptor's QE by that of its single microvillus, which is the ratio between its QB rate (*v*) and its photon‐absorption (*λ*) rate. This photon‐absorption rate is the light intensity divided by the number of microvilli, $\lambda =\frac{E(I)}{N_{\mu }}$, while the average QB rate of a microvillus is the reciprocal of its mean inter‐QB‐interval, *E*(*T*_*k*_). As the photon arrivals (absorption) are stochastic, the inter‐QB‐interval is also a stochastic variable, in which expectation can be calculated from its distribution.

(4) $$\text{QE}=\frac{N_{\mathrm{QB}}}{N_{ph}}=\frac{N_{\mathrm{QB}}/Tt}{N_{ph}/Tt}=\frac{N_{\mathrm{QB}}/Tt/N_{\mu }}{N_{ph}/Tt/N_{\mu }}=\frac{v}{\lambda }=\frac{\frac{1}{E(T_{k})}}{\frac{E(I)}{N_{\mu }}}$$

In Eq. 4, all the variables are known quantities, except the inter‐QB‐interval expectation, *E*(T_k_). Thus, we will first derive *T*_k_'s distribution. For analytical convenience, we also define other relevant stochastic variables:

H_k_ denotes the successive photon arrival intervals (*H*_k _= *t*_k _+ 1‐*t*_k_). Because {*t*_k_} is a Poisson point process, {H_k_} are i.i.d. random variables with the same exponential probability density functions (p.d.f.) that are independent of *k* (Miller 1965): $f_{I_{k}}\left(t\right)=\lambda e^{-\lambda t}.$

*T*_k_ denotes the inter‐QB‐intervals (*T*_k_=*b*_k‐1_‐*b*_k_). We assume that *T*_k_ are i.i.d random variables with the same p.d.f.

*L*_k_ is the latency (delay) between a photon arrival and its QB emergence. We assume that *L*_k_ follows a gamma distribution.

*D*_*k*_ is QB duration. In reality, *D*_*k*_ should be a stochastic variable, but here we assume that *D*_*k*_ has a fixed value.

*R*_k_ is the refractory period after a QB. We assume that *R*_k_ also follows a gamma distribution.

S_k_ is the minimum inter‐QB‐interval when the photons arrive before the RP termination:*S*_k _= *L*_k _+ *D*_k _+ *R*_k_.

*I*_*k*_ is the waiting time until the next photon arrival. Because {*t*_k_} is a Poisson point process, I_k_ has the same exponential probability density function with *H*_k_ (Poisson thinning property (Miller 1965)): $f_{I_{k}}\left(t\right)=\lambda e^{-\lambda t}.$ *A*, represents the events when *H*_k _> *S*_k_.

The steady‐state relationship between *I*_n_(*t*) and *C*_*n*_(*t*) can be alternatively studied from {*H*_*K*_} and {*T*_K_}; similar to {*t*_k_} and {*b*_k_}. Because here we are interested in the steady state analysis, we ignore the transformation from *t*_1_ to *b*_1_.

Figure 2 illustrates the relationships between the stochastic variables. Notably, the photon arrival intervals can either be larger or smaller than the minimum inter‐QB‐intervals. We use *A* to represent the event when the photon arrival interval is larger than the minimum inter‐QB‐interval (H_k_ > S_k_). The inter‐QB‐interval values, *T*_k_, have to be calculated differently, depending on whether *A* happens. When the event *A* is true (H_k _> S_k_), *T*_k_ can be approximated by the photon intervals (*T*_k _= *H*_k_). Since another QB cannot be excited during a QB, the effective next photon arrivals must be after *L*_k+_ *D*_k_ (*I*_*2*_ starts directly after *D*_*2*_). Because the *T*_k_ distribution is memoryless, *T*_k_ is *L*_k _+ *D*_k _+ I_K_. Conversely, when the event $\overset{¯}{A}$ is true (H_k_ < S_k_), photons that arrive during *S*_k_ cannot evoke QBs and will be lost (Fig. 2, 2nd blue dot). Under these circumstances, the next effective photon arrivals are after *S*_k _= L_k _+ D_k + _R_k_ (*I*_*1*_ starts after *R*_*1*_), and *T*_k_ is the summation of *S*_k_ and *I*_k_. Eq.5 formulates *T*_k_:

(5) $$T_{k}=\left\{\begin{array}{c} L_{k}+D_{k}+I_{k}\\ L_{k}+D_{k}+R_{k}+I_{k} \end{array}\right.,\begin{array}{c} \text{if}\;\begin{array}{cc} A: & H_{k}>S_{k} \end{array}\\ \text{if}\;\begin{array}{cc} \bar{A} & H_{k}<S_{k} \end{array} \end{array}$$

We then further assume that I_k_ *, L*_*k*_ *, H*_*k*_ *, R*_*k*_ are statistically independent for 0 < k<∞, and their respective p.d.f.s are all independent of *k*. Thus, *f*_*T*_(*t*), the p.d.f. of *T*_K_, can be derived as:

(6) $$f_{T}\left(t\right)=f_{L+D+I}\left(t\right)F\left(A\right)+f_{S+I}\left(t\right)\left(1-F\left(A\right)\right)$$

where f_s+I_(t) is the p.d.f. for the sum of *S* and *I. S + I* has the p.d.f.:

(7) $$f_{S+I}\left(t\right)=f_{S}{\left(t\right)}^{*}f_{I}\left(t\right)=\int_{0}^{t}f_{S}\left(t-\tau \right)f_{I}\left(\tau \right)d\tau$$

which is a convolution between *f*_*S*_(*t*) and *f*_*I*_(*t*). *f*_*s*_(*t*) can be calculated as the convolution between *f*_*L*_(*t*), *f*_*D*_(*t*) and *f*_*R*_(*t*): *f*_*s*_(*t*)=*f*_*L*_(*t*)*f_*D*_(*t*)*f_*R*_(*t*). Likewise, *f*_*L*+*D*+*I*_(*t*) is the convolution between *f*_*L*_(*t*)*, f*_*D*_(*t*)*,* and *f*_*I*_ *(t)*:* f*_*L+D+I*_(*t*)*= f*_*L*_(*t*)*f_*D*_(*t*)*f_*I*_(*t*). For simplicity, we ignore the stochastic QB waveform variations by assuming that the QB waveform *B*(*t*) is invariable. Then the p.d.f for *D* (QB duration) is the Dirac delta function that is zero except at value D: *f*_*D*_(*t*) = *δ*(*t* ‐ *D*)

Both *H* and *I* follow exponential distributions, with the same p.d.f. (Poisson thinning properties): *f*_*H*_(*t*) = *f*_*I*_(*t*) = *λe* ^−*λt*^. We further assume that both *L* and *R* are gamma‐distributed, *Γ*(*a*, *b*), with the corresponding p.d.f.:

(8) $$f\left(t,a,b\right)=\frac{b^{a}t^{a-1}{\text{e}}^{-bt}}{\Gamma (a)}t,a,b>0$$

where *a* and *b* are the shape and rate parameters, respectively. Eq. 8 defines a QB waveform, *B*(*t*). The parameters for defining *B(t)*,* f*_*L*_ *(t)* and *f*_*R*_ *(t)* (Table 1) were chosen to be physiologically realistic for mimicking a *Drosophila* photoreceptor's QB dynamics (Song et al. 2012).

**Table 1:** Parameters for QB waveforms, Latency and Refractory period (RP) distributions

	*B*(*t*)	*f*_L_(*t*)	*f*_R_(*t*)
*a*	9	9	9
*b*	1	3	8

*F(A)* in Eq.6 is the probability for the event *A*, and it can be calculated with Eq. 9:

(9) $$\begin{array}{cc} F(A) & =F(H>S)\\ & =\int_{0}^{\infty }\int_{S}^{\infty }f(H,S)dHdS\\ & =\int_{0}^{\infty }\int_{S}^{\infty }f(H)f(S)dHdS\\ & =\int_{0}^{\infty }f\left(S\right)dS\int_{S}^{\infty }f(H)dH\\ & =\int_{0}^{\infty }f\left(S\right)\left[1-F_{H}\left(S\right)\right]dS. \end{array}$$

where *F*_*H*_(*S*) is the probability for event *H* ≤ *S*. *H* follows an exponential distribution (*f*_*H*_(*t*) = *λe* ^‐*λt*^), *F*_*H*_(*S*) = 1 ‐ *e* ^‐*λS*^.

**Figure 3:**
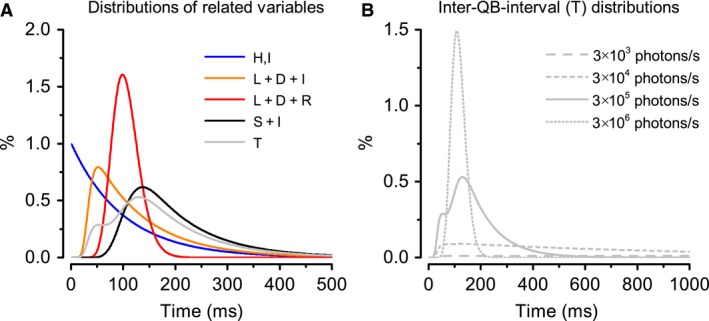
Inter‐QB‐interval distributions. (A) Probability distributions for different random variables defined. Both the photon intervals, H, and the next photon arrival waiting time, *I*, follow exponential distributions (blue line). L + D + I is the inter‐QB‐interval without a refractory period, but with a constraint that another QBcannot be excited before a QB termination (orange line). *S* = *L* + *D *+ *R* adds the physical constraint in QB generation; a second QB can only be triggered after the first QB's refractory period (red line). If photon arrival statistics is considered, *S* + *I* is the inter‐QB‐interval at bright light conditions (black line). Finally, the p.d.f. of inter‐QB‐intervals, T, is the weighted sum from the distributions of *L* + *D* + *I* and *S* + *I*. Because of this weighted operation, a hump emerges in T's p.d.f. at small inter‐QB‐intervals (light grey line). (B) Adaptation takes place in inter‐OB‐interval distributions at different light intensities.

From Eqs (5), (6), (7), (8), (9), we can calculate the inter‐QB‐interval distribution *f*_*T*_(*t*). To illustrate how *f*_*T*_(*t*) compares with the other distributions in deriving *f*_*T*_(*t*), Figure 3A shows the p.d.f.s for the different random variables at the light intensity of 3 × 10^5^ photons/sec per photoreceptor. It is interesting to notice how the inter‐QB‐interval distribution (light grey line) emerges from the photon arrival interval distribution (blue line).

Both the photon intervals, *H*, and the next photon arrival waiting times, *I*, follow exponential distributions. The mean arrival rate, *λ*, is calculated as the ratio between the number of incoming photons and the rhabdomere's microvillus count (*λ* = *N*_*ph*_/*N*_*μ*_)(Song et al. 2016).

*L*+*D*+*I* gives the inter‐QB‐interval without a refractory period, but with a constraint that another QB cannot be excited before the previous QB terminates (orange line). Its distribution is convolved from an exponential distribution and a gamma distribution, where the long tail shows the exponential distribution component.

*S *= *L *+ *D *+ *R* (red line) adds the QB generation constraints when the event $\overset{¯}{A}$ is true; a second QB can only be triggered after the first QB's refractory period. *S* is the inter‐QB‐interval when there are continuous photon arrivals, thus it represents the lower bound of the inter‐QB‐intervals under extreme brightness. In a *Drosophila* photoreceptor, *S* is dominantly determined by *R* (~100 msec), which is typically much longer than *L* and *D*.

For the photon arrival statistics at the brightest light condition, *S *+ *I* (black line) is the inter‐QB‐interval distribution; with the exponential *I* distribution contributing to the corresponding p.d.f.'s characteristic long tail.

Finally, for the presumed normal case at intermediate light conditions (Fig. 3A, light grey line), the p.d.f. of the inter‐QB‐intervals, *T*, as the weighted sum of *L *+ *D *+ *I* and *S *+ *I* distributions, shows a hump at small inter‐QB‐interval values.

Eq. 6 indicates that the inter‐QB‐interval distribution adapts with brightening, as shaped by the light input statistics and the refractory period, respectively. The weighting parameters are determined by the event *A* (*H > S*) probability, which changes with light conditions. In dim conditions, the probability for *A* is nearly one (100%), and the inter‐QB‐intervals predominantly reflect the photon arrival intervals (Fig. 3B, dash and short‐dash lines), with minimal RP contributions. But with brightening, the probability of *A* decreases, reducing the light input while increasing RP contributions so that the inter‐QB‐interval distribution loses its exponential long tail, approaching a gamma‐distribution at full daylight (Fig. 3B, short‐dot line).

**Figure 4:**
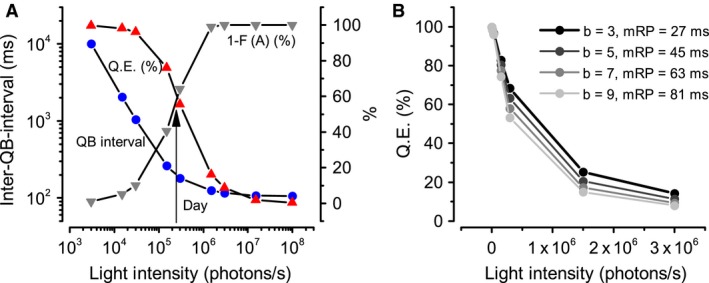
Inter‐QB‐intervals and QE adapt with brightening. (A) With increased contribution from refractory period (grey), the inter‐QB‐interval and QE reduces nonlinearly with brightening (blue and red). In dim light conditions, the mean photon arrival intervals can approach to 10 sec, which is much longer than the mean RPs (~100 msec). In these cases, refractory period contributes minimal, and QE approaches 100%. As light intensity increases, inter‐QB‐interval drops roughly linearly until an intensity that matches the population QB rate (QB rate/microvillus x #microvilli). In this particular case for a Drosophila photoreceptor, this light intensity is 3 × 10^5^ photons/sec, replicating living room day light conditions. It is also at this light condition that RP start to play an important role in tuning the system's gain (contributions over 50%), leading a sharp drop in the QE. As it becomes brighter, inter‐QB‐interval approaches to its limit, where RP is the dominating factor (>90%). The QE drops to 8% at the outdoor overcast light intensity (3 × 10^6^ photons/sec). When the light intensity increases to 1 × 10^6^ photons/sec (bright daylight), the QE can even drop to 0.26%. (B) The reduction rate of QE versus brightening is highly dependent on the statistical properties of refractory periods. We only tuned parameter b for the gamma distribution of refractory periods, and kept parameter “*a*” the same as shown in Table 1. With increasing *b*, the mean of refractory periods (mRP) increases, and the rate of QE reduction goes up.

Using these inter‐QB‐interval distributions (Fig. 3B), we calculated the inter‐QB‐interval expectations and QE at different light intensities (Fig. 4A). Both decrease nonlinearly with brightening. In dim conditions, photon arrivals are so sparse that the mean photon arrival intervals approach 10 sec, which is much longer than the mean RPs (~100 msec). In these cases, the QE nears 100% (all the absorbed photons are transduced to QBs), and the RP does not affect encoding. With brightening, the inter‐QB‐interval reduces roughly linearly until the intensity matches the population QB rate (QB rate/microvillus x #microvilli). For a *Drosophila* photoreceptor, the QE falls sharply at this light intensity (3 × 10^5^ photons/sec ~brightly illuminated room) as the RP starts to affect the system's gain considerably (contributing > 50%). With further brightening, the inter‐QB‐interval approaches its limit and the RP becomes the dominating factor (>90%). The QE falls to 8% at the outdoor overcast light intensity (3 x 10^6^ photons/sec). When the light intensity is 1 × 10^8^ photons/sec (bright day shade), the QE falls to 0.26%. Notice, however, that because of the intracellular pupil mechanism and other photomechanical adaptations (Juusola et al. 2016; Juusola and Song 2017), a *Drosophila* photoreceptor would likely never directly face this intense light. Nonetheless, these simulations are useful in defining the bounds for how much the RP can limit the QB production rate at daylight conditions, suggesting that a ~500‐fold gain reduction would be possible. Thus, by losing photons with brightening to refractory microviili, QE is reduced automatically, enlarging the system's dynamic range.

Predictably, a photoreceptor's gain regulation depends upon the statistical refractory period properties. To show this, we tuned parameter “*b*” for the RP gamma distribution, and kept “*a*” the same as in Table 1. With increasing *b*, the mean of refractory period (mRP) increases, and QE reduces quicker (Fig. 4B).

**Figure 5:**
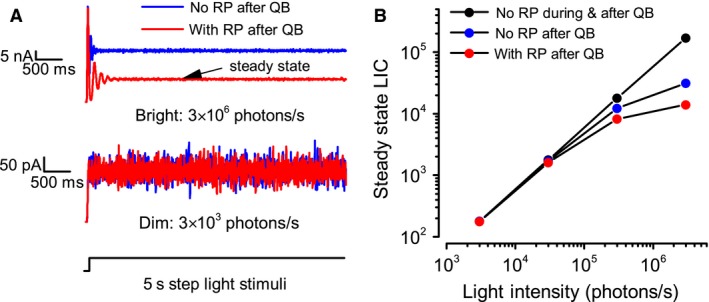
Comparison of steady state LIC, modeled with and without RP. (A) Simulated LIC with and without RP after QB. 5 sec light step was the input (bottom), and the mean steady state LIC the output. In dim light (middle), RP plays no role, and the LICs with and without RP have similar amplitudes. In bright light (top), RP reduces the steady state response by half, tuning the system gain nonlinearly. (B) If all photons were converted to a stereotyped QB, the Intensity‐LIC relationship would be linear (black). However, with photons lost during the QB, the intensity‐LIC relationship reduces at bright conditions (blue). With a further RP after QB, the gain reduces more with brightening (red).

Finally, we mention that photons can be lost over two periods: during a QB and in the following refractory period. To further quantify how these losses affect the steady state response, we compared the simulated LICs with and without RP to a 5 sec light step (Fig. 5A, bottom). In dim light (Fig. 5A, middle), RP plays no role, and the LICs with and without it have similar amplitudes. In bright light (Fig. 5A, top), RP reduces the steady state LIC response by half, tuning the system gain nonlinearly. Thus, if all photons were transduced to a stereotyped QB, the Intensity‐LIC relationship would be linear (Fig. 5B black). But with photons lost during the QB, the intensity‐LIC relationship reduces (Fig. 5B, blue), and with a RP lasting over the QB, the gain reduces even more with brightening (Fig. 5B, red).

## Discussion

We tested by modeling how refractory period can impact sampling and integration of quantal events in a prototypical graded potential neuron. The simulations were run by a simple stochastic process model for the fly phototransduction, which combined probabilistic photon‐QB conversions with a RP after each QB. We then derived the inter‐QB‐interval distributions and quantified the system's gain by calculating QE. Because the inter‐QB‐intervals were calculated differently, depending on whether the next photons arrived before or after the RP termination, the role of RP in adaptation became clear. The results quantified how RP provide an automatic gain control, the strength of which depends upon both the light stimulus and RP statistics.

Using physiologically realistic parameters for the relevant stochastic variables, our results imply that RP cannot affect encoding at dim light; with a fly photoreceptor maintaining 100% QE. But with brightening, RP reduces the QE nonlinearly. Theoretically, the QE could fall to 0.26% at very bright light, meaning that RP would have reduced the system's gain by 500‐fold. Thus, with the refractory microvilli discarding excess photons, the system's dynamic range is enlarged automatically and extensively (see also Song et al. 2012).

However, RP is not the only mechanism that helps to extend a photoreceptor's dynamic range. Other mechanisms include the intracellular pupil; QB size adaptations; variable QB latencies; nonlinear shunting by voltage‐gated conductances; and photo‐mechanical rhabdomere contractions (Juusola et al. 2016). These were ignored here, as our aim was not to replicate photoreceptor responses but to quantify how neural gain control can emerge from RP.

The relationship between RP and scaling in neuronal action potential encoding has been discussed in earlier studies. For example, how RP affects action potential firing in the cat retinal ganglion cells (Teich et al. 1978) and auditory nerve fibers (Li and Young 1993) has been analyzed from the digital spike‐code perspective. It was suggested that the spike generator in the ganglion cells could be the main retinal site where refractoriness shapes encoding (Teich et al. 1978). Interestingly, however, our recent (Song et al. 2012; Song and Juusola 2014) and new results imply that RP can already shape neural responses to visual stimuli at the first stage of light information sampling and processing ‐ well before any spike‐coding. Based on the profound benefits stochastic refractoriness provides for encoding fast and large stimulus changes, including amplitude normalization and antialiasing (Song et al. 2012; Juusola et al. 2015, 2016), we speculate that it, in fact, might be a generic biophysical sampling property for micro/nanodomain quantal events. For instance, theoretically, a large population of refractory mechanosensitive ion channels can rapidly and reliably reproduce the electrical behavior of a mechanoreceptor (Song et al. 2015). Thus, RP may not only affect neural firing, but its impact on encoding should also be considered in sub‐threshold signals.

Importantly, the refractory period in QB production differs mechanistically from that of the spikes. In the giant squid axon, the RP in action potential production originates from the recovery phase K^+^‐current (Hodgkin and Huxley 1952a), whereas the fly photoreceptors' RP is an intrinsic property of their microvillar phototransduction cascades, driven by Ca^2+^ feedbacks (Song et al. 2012). Furthermore, in spiking neurons, one can distinguish the absolute and relative refractory period by their different physiological properties. The absolute refractory period typically refers to the Na^+^‐channel inactivation period, during which a new spike cannot be triggered by any stimuli. But during the relative refractory period, when the inward Na^+^‐currents is shunted by opposing K^+^‐conductances, new spikes can be triggered by stronger stimuli. Conversely, the refractory period after a QB is always absolute; with QBs being all‐or‐none responses, there is no concept of “relative” RP.

In spiking neurons, the concept of gain control is characteristically viewed through Hodgkin‐Huxley (HH) formalism by modeling voltage‐gated conductances on the cell membrane. There, Shunting inhibition is considered the primary mechanism for adaptation (Blomfield 1974), with additional contributions from noise (Prescott and de Koninck 2003; Dunn and Rieke 2006), dendritic saturation (Prescott and de Koninck 2003) and synaptic inputs (Chance et al. 2002). However, in graded potential systems, many biophysical mechanisms shape the elementary quantal events adaptively (their shape, RP and stochasticity) (Song et al. 2012, 2015, 2016; Song and Juusola 2014; Juusola et al. 2015). These mechanisms are governed by biochemical networks upstream of spike production, and thus are concealed from HH‐models. More detailed biophysical approaches, both for computational simulations and theoretical analysis, are required to assess how adaptive quantal event dynamics affect the systems level gain control. We hope that our results could be valuable also for understanding adaptive dynamics in synaptic transmission and in network functions (Carrillo‐Medina and Latorre 2015). After all, signal integration from a large population of refractory synapses or refractory neurons is conceptually similar to the large microvillus population.

## Conflict of Interest

None declared.
